# Rationale and design of the “NEurodegeneration: Traumatic brain injury as Origin of the Neuropathology (NEwTON)” study: a prospective cohort study of individuals at risk for chronic traumatic encephalopathy

**DOI:** 10.1186/s13195-022-01059-8

**Published:** 2022-09-01

**Authors:** Suzan van Amerongen, Dewi K. Caton, Rik Ossenkoppele, Frederik Barkhof, Petra J. W. Pouwels, Charlotte E. Teunissen, Annemieke J. M. Rozemuller, Jeroen J. M. Hoozemans, Yolande A. L. Pijnenburg, Philip Scheltens, Everard G. B. Vijverberg

**Affiliations:** 1 Amsterdam Neuroscience, Neurodegeneration, Amsterdam, The Netherlands; 2 Alzheimer Center Amsterdam, Neurology, Vrije Universiteit Amsterdam, Amsterdam UMC location VUmc, Amsterdam, The Netherlands; 3 Brain Research Center, Amsterdam, The Netherlands; 4 Clinical Memory Research Unit, Lund University, Lund, Sweden; 5 Department of Radiology & Nuclear Medicine, Amsterdam UMC, location Vrije Universiteit Amsterdam, Amsterdam, The Netherlands; 6 UCL Institute of Neurology, London, UK; 7 Neurochemistry Laboratory, Department of Clinical Chemistry, Amsterdam UMC, location Vrije Universiteit Amsterdam, Amsterdam, The Netherlands; 8 Department of Pathology, Amsterdam UMC, location Vrije Universiteit Amsterdam, Amsterdam, The Netherlands

**Keywords:** Chronic traumatic encephalopathy, Repetitive head injury, Traumatic brain injury, Contact sports, Cognition, Neuropsychiatry, Cognitive decline, Magnetic resonance imaging, Fluid biomarkers, Neuropathology

## Abstract

**Background:**

Repetitive head injury in contact sports is associated with cognitive, neurobehavioral, and motor impairments and linked to a unique neurodegenerative disorder: chronic traumatic encephalopathy (CTE). As the clinical presentation is variable, risk factors are heterogeneous, and diagnostic biomarkers are not yet established, the diagnostic process of CTE remains a challenge. The general objective of the NEwTON study is to establish a prospective cohort of individuals with high risk for CTE, to phenotype the study population, to identify potential fluid and neuroimaging biomarkers, and to measure clinical progression of the disease. The present paper explains the protocol and design of this case-finding study.

**Methods:**

NEwTON is a prospective study that aims to recruit participants at risk for CTE, with features of the traumatic encephalopathy syndrome (exposed participants), and healthy unexposed control individuals. Subjects are invited to participate after diagnostic screening at our memory clinic or recruited by advertisement. Exposed participants receive a comprehensive baseline screening, including neurological examination, neuropsychological tests, questionnaires and brain MRI for anatomical imaging, diffusion tensor imaging (DTI), resting-state functional MRI (rsfMRI), and quantitative susceptibility mapping (QSM). Questionnaires include topics on life-time head injury, subjective cognitive change, and neuropsychiatric symptoms. Optionally, blood and cerebrospinal fluid are obtained for storage in the NEwTON biobank. Patients are informed about our brain donation program in collaboration with the Netherlands Brain Brank. Follow-up takes place annually and includes neuropsychological assessment, questionnaires, and optional blood draw. Testing of control subjects is limited to baseline neuropsychological tests, MRI scan, and also noncompulsory blood draw.

**Results:**

To date, 27 exposed participants have finished their baseline assessments. First baseline results are expected in 2023.

**Conclusions:**

The NEwTON study will assemble a unique cohort with prospective observational data of male and female individuals with high risk for CTE. This study is expected to be a primary explorative base and designed to share data with international CTE-related cohorts. Sub-studies may be added in the future with this cohort as backbone.

## Background

Traumatic brain injury (TBI) is a serious problem in healthcare. In the Netherlands, an estimated 30,000 patients yearly visit the emergency department because of TBI. The prevalence of TBI is even likely to be higher, given that this number does not include patient visits to a primary care doctor or patients with un- or misdiagnosed TBI [1, 2]. In most patients (80–90%), head injury is classified as mild TBI and concussive symptoms will resolve after several days or weeks [3]. A small percentage of patients experience persistent neurological symptoms (headache, dizziness, cognitive complaints) and may develop a post-concussion syndrome [4]. Not only is TBI related to acute and subacute neurological symptoms, it is also associated with long-term neurological consequences. Previous studies have found an association between a past medical history of TBI and higher risk of neurodegenerative diseases, such as Alzheimer disease (AD) [5, 6], Parkinson’s disease [7], frontotemporal dementia [8, 9], and amyotrophic lateral sclerosis [10]. Additionally, a higher mortality from neurodegenerative disease is found among former contact sports athletes, who are more likely to have experienced recurrent impacts to the head than the average population [11, 12]. These findings indicate that single or repetitive head injury is a risk factor for neurodegeneration later in life.

The established relationship between repetitive head injury and late-life cognitive impairment was already reported in the early twentieth century, when the terms “punch drunk syndrome” or “dementia pugilistica” were used to describe a neuropsychiatric syndrome in former boxers [13], nowadays better known as chronic traumatic encephalopathy (CTE). CTE is classified as a neurodegenerative disease, associated with a history of repetitive head injury and confirmed by post-mortem neuropathological assessment [14]. CTE is characterized by the accumulation of hyperphosphorylated tau (p-tau) in areas around small blood vessels at depths of the sulci in the cerebral cortex. To date, CTE pathology has been observed in former contact sport athletes (boxing, American football, ice hockey, soccer) and in military personnel, but the exact prevalence in these populations is unknown [15, 16]. The clinical manifestation of CTE is thought to be highly diverse, with a symptom onset between 30 and 60 years old and a range of impairments in cognition, behavior, mood, and motor function [17]. No consensus on clinical diagnostic criteria for CTE has been reached. In order to classify the clinical characteristics of CTE, renewed research criteria for the traumatic encephalopathy syndrome (TES) have recently been published [18]. However, these criteria have limitations, given that they were established based on mainly retrospective observational pathological studies and most symptoms were recorded during postmortem interviews with relatives, leading to a high risk of recall and selection bias. As CTE can solely be diagnosed by neuropathological examination, more insight is needed in the clinical manifestation of this disease.

Thus far, the exact role of in vivo biomarkers in the diagnostic process of CTE have remained unclear. Several fluid biomarkers in cerebrospinal fluid (CSF) or plasma may be promising, reflecting disease specific pathology (p-tau markers), neuronal injury (i.e. total tau or neurofilament light chain), and inflammation (C-C motif chemokine 11), but their diagnostic and prognostic value in CTE is unknown [19]. Besides, a variety of magnetic resonance imaging (MRI) abnormalities have been reported after repetitive head injury which are thought to be associated with CTE, particularly a cavum septum pellucidum, but also periventricular enlargement, cerebral atrophy, and white matter alterations [20–24]. Some researchers have also attempted to visualize tau depositions in CTE-related areas with flortaucipir positron emission tomography (PET) scans. One study demonstrated slightly higher tau-levels in a group of symptomatic former American Football players compared to controls, which was found in the bilateral superior frontal areas, bilateral medial temporal areas, and the left parietal area [25]. Another study revealed only mildly elevated tau-binding in brains of former symptomatic contact-sport athletes, limited to frontotemporal regions [26]. While potentially promising, current tau-tracers are probably not suitable to detect tau in CTE patients and more research is necessary to evaluate the potential role of tau-PET imaging. If in vivo detection in CTE is made possible in the future, it will offer more insight into the epidemiology, risk factors, and clinical progress of the disease.

To summarize, the link between repetitive head injury and neurodegeneration is undisputed. CTE has received increasing attention over the past decades, but uncertainties remain regarding the etiology, clinical presentation, course and detection of CTE during life. The “*NE*urodegeneration, *T*raumatic brain injury as *O*rigin of the *N*europathology” (NEwTON) study addresses these gaps in literature by further investigating CTE and unraveling the link between repetitive head injury and neurodegeneration. The NEwTON study aims to:

- Identify clinical and cognitive characteristics of individuals at risk for CTE
- Measure the clinical course of individuals at risk for CTE
- Identify potential diagnostic and prognostic biomarkers in CSF, blood, or with MRI in individuals at risk for CTE
- Recruit potential candidates for autopsy brain donation to the Netherlands Brain Bank in order to investigate post-mortem pathology of individuals at risk for CTE
- Exchange data with international institutions involved in CTE research

The present paper explains the protocol and design of this prospective case-finding study.

## Methods

### Study infrastructure

**Fig. 1:**
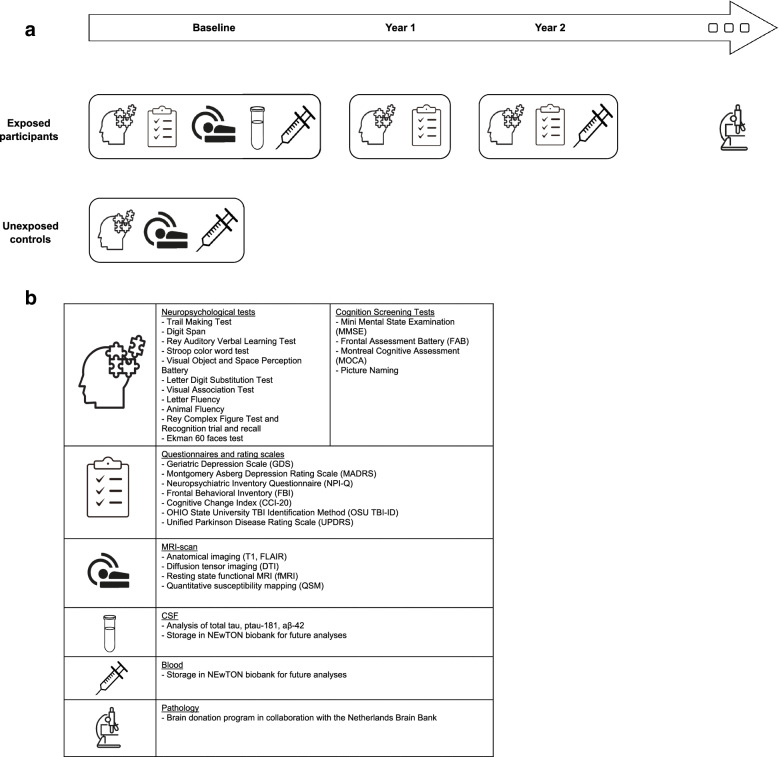
**a** Study infrastructure of NEwTON. **b** Overview of all study procedures within the NEwTON protocol

NEwTON is a single-center observational prospective case-finding study based at the Alzheimer Center Amsterdam that aims to include 40 participants at risk for CTE (exposed participants) and 40 healthy unexposed control subjects. Exposed participants and unexposed controls are allocated to various study procedures. All exposed participants receive a comprehensive baseline screening and are invited for two follow-up measurements (after 1 and 2 years). If the included participant comes from the memory clinic and has received standard diagnostic screening less than 6 months before inclusion in the NEwTON study, only additional measurements are done, because part of the study procedures overlap with the standard diagnostic screening. The procedure for control subjects is limited to one baseline assessment, including a neuropsychological test battery, blood draw, and MRI scan of the brain (Fig. 1a). The rationale behind a single baseline assessment for healthy controls is to diminish participation burden for this population. Besides, the control group is included in the study to identify, validate and compare potential neuro-imaging/fluid biomarkers, which are mainly measured at baseline.

### Population

#### Participant characteristics

The inclusion criteria for *exposed participants* are derived from the criteria for TES by Katz et al. [18]:

1. History of repetitive/multiple impacts to the head. Sources of exposure could be:
  ◦ Involvement in high exposure contact sports (i.e. combat sports, rugby, soccer) for a minimum period of 6 years on significant level
  ◦ History of any other significant exposure to repetitive hits on the head (i.e. abuse, head banging behavior, military service)
  ◦ Any other activity resulting in multiple TBI (fall, traffic accident)
2. At least one of the following core clinical features of TES must be present and be different from pre-morbid functioning. Symptoms may be self-reported, reported by informant, reported by clinician’s report or objectified by previous standardized clinical testing
  ◦ Cognitive symptoms: memory, executive functioning
  ◦ Neurobehavioral dysregulation: emotionally explosive, physically/verbally violent, “having a short fuse”
3. Clinical features must be present for a minimum of 12 months
4. Age above 30 years old

Important to note is that participants with a previous diagnosis of any other neurological or psychiatric disease are also able to participate.

Inclusion criteria for *unexposed controls* are as follows:

1. Age above 30 years old
2. No history of participation in organized contact/collision sports*
3. No history of military service (professional, with blast exposure)
4. No history of clinically significant TBI or concussion
5. No history of any neurological, psychiatric or neurodegenerative disease
6. No reported complaints of cognition, behavior and depressive mood

*This is indicated as no history of organized participation in any of the following sports: soccer (under age 14 allowed if no significant heading the ball), rugby, boxing, kickboxing, Muay Thai, Mixed Martial Arts, American football, ice hockey, lacrosse, and wrestling.

**Fig. 2:**
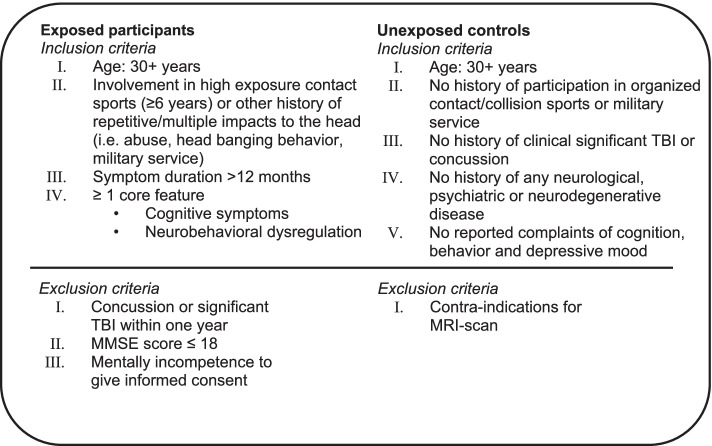
Inclusion/exclusion criteria for participation in the NEwTON study

The exclusion criteria for participation include an insufficient knowledge of the Dutch language or mentally incompetency to give informed consent. Furthermore, exposed participants are excluded with a Mini-Mental State Examination (MMSE) score of ≤ 18 or when they have reported a clinically significant concussion or traumatic brain injury within 1 year before inclusion. Control subjects are excluded when there is a contra-indication for MRI according to the hospital protocol. An overview of the participant criteria is given in Fig. 2.

#### Recruitment

Several sources of recruitment are used to find eligible participants for NEwTON. Part of the participants are recruited from the memory clinic of the Alzheimer Center Amsterdam, an expertise center in dementia. As part of the standard diagnostic work-up in the Alzheimer Center Amsterdam, patients receive standardized diagnostic tests. Patients are asked to sign a consent form indicating that their clinical information can be used for scientific purposes in the future and that they may be approached for further research participation. If patients fulfill criteria and they are interested in participating, they are invited to undergo additional measurements for the NEwTON study at a later stage.

Other potentially eligible participants are recruited outside the Alzheimer Center’s memory clinic. The NEwTON study has already established collaborations with different national sports federations, such as the Dutch Olympic Committee*Dutch Sports Federation (NOC*NSF), the national soccer union (“Koninklijke Nederlandse Voetbalbond”), the national rugby federation (“Rugby Nederland”), the national combat sports authority (“Nederlandse Vechtsport Autoriteit), and the Dutch Boxing Federation (“Nederlandse Boksbond”). NEwTON is able to promote its study by utilizing their media platforms to reach an eligible audience. Unexposed controls are reached by the media platforms of the Alzheimer Center Amsterdam. Potential candidates are able to contact the researchers via e-mail. When eligible, they are invited to participate to the NEwTON study and undergo all baseline measurements.

#### Power

For this study, it is complicated to determine a study sample size that achieves adequate statistical power. This is mainly because NEwTON has an exploratory research design, with various outcome measures that have not been well-studied yet in previous research. To illustrate, previous results on fluid and neuro-imaging biomarkers are preliminary and have not been tested in a similar study population. Additionally, there is no available prospective data on clinical progression of CTE. The sample size of 40 exposed participants and 40 unexposed controls is considered sufficient for a pilot study and this number is expected to be expanded in the future. This is in line with the decision of the current 2-year follow-up period, which also may be extended in the future to make it possible to measure the clinical course over a longer period of time

### Study procedures

An overview of all study procedures is displayed in Fig. 1b.

#### Clinical examination

All exposed participants receive a structured medical interview by a trained physician, to collect data about demographics, neurological symptoms, previous medical history, medication, current or previous substance use, lifestyle history, and family history of any neurological or psychiatric diseases. In addition, a comprehensive sports history assessment is performed to identify previous participation in (contact) sports and to collect information about age, era and duration of participation, position played, the highest level of competition, and the use of head protection equipment. If the participant consents, his or her informant is also interviewed. Vital signs are administered and neurological examination is performed. Motor symptoms are measured according to the motor score of the unified Parkinson’s disease rating scale (UPDRS), a standardized rating scale to score extrapyramidal signs. These examinations are repeated at follow-up screening.

#### Neuropsychological assessment

The MMSE and Montreal Cognitive Assessment (MOCA) are both administered to screen global cognitive functioning. Although there is some overlap in the content of these tests, having the results of both tests in the data set will be of high value for future national and international data exchange. The Frontal Assessment Battery (FAB) and a picture naming test are also included in the protocol, which are screening tests to assess frontal lobe dysfunction and language problems respectively. Additionally, all participants undergo a comprehensive neuropsychological test battery, that includes tests for different cognitive domains: Trail Making Test Part A & B *(processing speed, sequencing, mental flexibility and visual-motor skills)*, Stroop Color Word Test and Digit Span *(attention and inhibition)*, Letter Digit Substitution Test *(visual scanning, mental flexibility, sustained attention, psychomotor, and processing speed)*, Verbal Fluency Test *(lexical memory, executive functions)*, Animal Fluency Test *(semantic memory)*, Visual Association Test and Rey Auditory Verbal Learning Test *(visual and verbal memory)*, Rey Complex Figure Test and Recognition trial and recall *(visuospatial constructional ability and visual memory)*, Visual Object and Space Perception Battery *(visuospatial constructional ability)*, Dutch Reading Test *(premorbid intelligence level),* and Ekman 60 faces test *(social cognition).* This test battery is applied again to exposed participants after one and two years.

#### Subjective cognitive impairment and mental health

Multiple questionnaires and rating scales are included for exposed participants in NEwTON regarding subjective cognitive impairment and mental health:

- The Cognitive Change Index—20 item (CCI-20): this questionnaire has two versions (self-report and informant report), both with 20 questions that reflects subjective cognitive change compared to 5 years ago. Each item is scored from 1 (no change) to 5 (very severe decline).
- Geriatric Depression Scale (GDS)—15-item: a questionnaire to test depressive symptoms in the elderly. Each question is answered by yes or no and corresponds to a positive or negative indication of depression. Higher scores reflect more severe depressive symptoms
- The Montgomery Asberg Depression Rating Scale (MADRS): this 10-item rating scale is completed by the physician after the clinical interview with the participant and reflects clinical judgment on different depressive symptoms. Every item is rated from 0 to 6, with higher rates indicating more severe symptoms.
- The Neuropsychiatric Inventory Questionnaire (NPI-Q): this questionnaire is completed by the informant and administered by a trained researcher via a face-to-face interview or telephone call. Twelve different neuropsychiatric symptoms are measured (delusions, hallucinations, agitation/aggression, dysphoria/depression, anxiety, euphoria/elation, apathy/indifference, disinhibition, irritability/lability, aberrant motor behavior, night-time behavior and appetite/eating). The researcher tests whether the symptom is present and, if so, measures the frequency (range 1–4), severity (range 1–3), and emotional distress that the symptom causes (range 1–5).
- The Frontal Behavioral Inventory (FBI): this 24-item inventory assesses behavioral changes and was developed to test symptoms of the behavioral variant of frontal temporal dementia but is administered in this study because current research lacks validated tools to assess CTE-related behavioral changes. The informant rates every item from 0 (not present) to 3 (severe).

#### Traumatic brain Injury (TBI)

At baseline, a comprehensive assessment of TBI history is applied to exposed participants by means of a translated version of the OHIO State University TBI Identification Method (OSU TBI-ID). The aim of this method is to reveal the life-time history of TBI and comprises three parts. The first part contains questions to identify and recall TBI in history. The second part aims to classify each TBI (cause, age, loss of consciousness or amnesia) and part three identifies any periods in life with repetitive head impacts (for example in contact sports). For each period, the age and cause of the head impacts are listed, as well as the typical effect and the most severe effect of the impacts during this period. In addition, concussion related symptoms are measured by items of the Rivermead Post-Concussion Symptoms Questionnaire.

#### MRI

MRI scans are acquired on a GE 3-Tesla scanner (Discovery MR750) for both exposed participants and unexposed control subjects, with approximately 40 min acquisition time. High-resolution (1 × 1 × 1 mm^3^) T1-weighted imaging and fluid-attenuated inversion recovery (FLAIR) are used for anatomical imaging. Diffusion-weighted images (DWI) are acquired to determine white matter integrity, with a resolution of 2 × 2 × 2 mm^3^ and 2 different *b* values (5 b0 and 30 volumes with b1000 s/mm^2^). Resting-state functional MRI (fMRI) is used to determine brain network connectivity, measured by the degree of synchronized blood-oxygenation-level dependent (BOLD) signal across brain regions. The T2*-weighted echo-planar images are acquired with 3.3 mm^3^ resolution. With quantitative susceptibility mapping (QSM), a 3D gradient echo sequence is used to determine spatial distribution of magnetic susceptibility. This is sensitive to presence of iron, calcium, and myelin in the brain. QSM images are acquired using a 3D multiple echo gradient echo sequence at resolution 0.5 × 0.5 × 1.6 mm^3^. Reference scans with reversed phase-encode direction are acquired for fMRI and DWI, to allow correction of susceptibility induced distortion. For fMRI, DWI and QSM, high-order shimming is performed before each scan.

Prior to the analyses, FLAIR imaging is used to detect white matter hyperintensities and cortical lacerations, which will be corrected for in further evaluations. FreeSurfer is used to measure structural brain features from T1 images, including grey and white matter volume, hippocampal volume, and cortical thickness. Additionally, the presence and width of cavum septum pellucidum is measured. For DTI data, FMRIB Software Library (FSL) tools are used for pre-processing, with correction for susceptibility-induced distortion and motion. Four metrics are estimated to identify white matter damage. (1) fractional anisotropy, the most common parameter to detect differences in white matter; (2) mean diffusivity, the diffusivity averaged over all directions; (3) axial diffusivity, which is sensitive to diffusivity along the axon; and (4) radial diffusivity, to determine diffusivity parallel to the axons. For group comparisons, the DTI ToolKit (DTI-TK) is used, which is a spatial normalization and atlas construction toolkit, that takes into account the main diffusion direction. The fMRI images are pre-processed and analyzed using the FSL toolbox. QSM data is visually analyzed for cerebral microbleeds and siderosis and maps are calculated with the help of the online toolbox: Sepia (SuscEptibility mapping PIpeline tool for phAse image), based on the pipeline that has been developed for previous QSM projects using similar input data [27]. For these analyses, pre-defined region-of-interests, white matter tracts, and functional brain networks will be examined, informed by the current literature. In addition, exploratory voxel-wise analyses will be performed to examine more fine-grained regional change.

#### Body fluid biomarkers

Participants are asked to give separate consent to the researchers for blood collection, CSF collection, and storage of these body fluids in the NEwTON biobank for future analysis. However, this is not mandatory for participation in the study.

##### Venous blood

Venous blood is drawn by a trained physician or researcher at baseline (both exposed participants and controls) and at 2-year follow-up (exposed participants only). The plasma and serum samples (24 ml in total) are centrifuged (1800 g, 10 min) at room temperature within 2 h after collection, aliquoted into small vials (0.5 ml) and stored at − 80 °C at the NEwTON biobank located at the clinical chemistry laboratory of the Amsterdam University Medical Centers (UMC) [28]. In addition, buffy coat is isolated after centrifugation and stored separately to purify DNA in the future.

##### CSF

All exposed participants are invited to undergo a lumbar puncture (LP), which is performed by a neurologist or trained physician after subject’s consent. Contra-indications for LP are determined according to local hospital guidelines and participants are informed about the procedures and potential complications [29]. After collection, a small amount of CSF is used for routine analysis on white blood cells, erythrocytes, proteins, and glucose. Amyloid beta (aβ) 1-42, total tau, and p-tau-181 are determined by using Elecsys® assays. A separate portion of CSF is centrifuged (1200 g, 10 min, room temperature) within 2 h and aliquoted into small vials and stored at − 80 °C in the NEwTON biobank [30].

#### Brain donation program

The NEwTON study has established a national CTE brain donation program in collaboration with the Netherlands Brain Bank (NBB). Former contact sport athletes are able to register as brain donor at the NBB thus consent for post-mortem brain autopsy. All participants of NEwTON are informed about this donation program and the possibility to register. Tissue treatment, sample storage, and pathological evaluation of NEwTON brain donors is performed according to standardized protocol of the NBB, including macroscopic evaluation and immunohistochemical staining of multiple brain regions: hematoxylin and eosin (H&E stain), aβ stain, Gallyas, silver-staining, several tau staining (AT8, RD3, RD4), alpha-synuclein, and TAR DNA-binding protein-43 (TDP-43). Neuropathological diagnosis will be established according to international guidelines of Brain Net Europe II (BNE) consortium and NIA-AA criteria for AD neuropathological change [31, 32]. Brain tissue is stored frozen or formalin fixed and embedded in paraffin at the NBB for future research purposes.

#### Multidisciplinary consensus meeting

Every exposed participant is discussed in a multidisciplinary consensus meeting that is attended by at least one neurologist and one neuropsychologist. During this meeting, a consensus diagnosis is made for each participant regarding the TES criteria by Katz et al. [18]. The panelists also determine the probability of CTE according the TES flow-chart of this study and the disease stage (subjective cognitive decline, mild cognitive impairment or dementia) for each exposed participant.

### Statistics

All statistical analyses of the data will be performed with IBM SPSS Statistics or R Studio. Within exposed participants, explorative analyses are performed to assess associations between cumulative head injury exposure, and cognitive/mental health outcome measures, body fluid biomarkers, and MRI data. Correlation coefficients and linear regression analyses are utilized to test these associations, including adjustment for potential confounders. Furthermore, the progression of cognitive and mental health outcome measures in participants over time is assessed using linear mixed models. For group comparisons, neuropsychological test results, blood biomarkers and MRI data are compared at baseline between groups (exposed participants and unexposed controls) by independent *T*-tests, analyses of variance (ANOVA), or Mann-Whitney *U* test, where appropriate.

### Ethical consideration and data sharing

All research conducted by NEwTON is in accordance with the World Medical Association (WMA) Declaration of Helsinki, Ethical Principles for Medical Research Involving Human Subjects 2013 and has been reviewed by the Medical Ethics Committee from the Amsterdam UMC. Before inclusion, all participants sign written informed consent, including separate permission to store body fluids in the NEwTON biobank for future analysis. Furthermore, explicit consent is obtained from all participants regarding sharing data and/or biomaterials with institutions from abroad, including countries outside the European Union. To illustrate, preliminary collaborations are made with the Boston University CTE Center and the “Diagnostics, Imaging, and Genetics Network for the Objective Study and Evaluation of Chronic Traumatic Encephalopathy” (DIAGNOSE CTE) Project for data sharing in later phases of the project. Handling and sharing of data and/or biomaterials will be in agreement with the General Data Protection Regulation (GDPR) or at the best possible level of confidence, when different regulations apply, such as in the USA. All data and/or biomaterial will be shared under a transfer/sharing agreement.

## Results

At June 1, 2021, the Medical Ethics Committee gave approval to NEwTON to launch the study. The first participant was included at July 15, 2021, and thus far, 27 exposed participants have finished their baseline assessments. It is to be expected that the first baseline results will appear in 2023.

## Discussion

This paper provides detailed information about the objectives and design of the NEwTON study, which is a prospective case-finding study including subjects that are at risk for CTE. These subjects have been exposed to repetitive head impacts and experience cognitive complaints and/or changes in behavior. The aim of this study is to identify clinical and cognitive characteristics of CTE, to find disease-related biomarkers, and to compare these outcome measures with unexposed control subjects. A comprehensive overview was given on the participant selection, recruitment methods, and the study procedures at baseline and follow-up.

The long-term consequences of repetitive head injury have received growing attention previous years and the awareness of this problem has led to the establishment of multiple prospective cohort studies worldwide. For example, Boston University CTE Center is performing a research project with similar aims to NEwTON’s: the DIAGNOSE study, which focuses on former American Football Players [33]. Similarly, the University of Glasgow’s “BRAIN” and HEADING” projects investigate clinical and cognitive outcomes measures in former rugby players and soccer players, respectively [34, 35]. However, all these cohorts concentrate on one sport only. Furthermore, the study designs from Glasgow do not focus on CTE in particular and their designs are limited to cross-sectional analysis. NEwTON is the first prospective cohort of patients at risk for CTE in the Netherlands and is unique due to its focus on the identification of CTE in multiple contact sports athletes such as (kick)boxing athletes, MMA fighters, soccer players, American Football players, and rugby players. Another difference compared to previous cohorts is that the NEwTON study includes a heterogeneous sample with male and female athletes. These unique features of NEwTON’s study protocol will increase the understanding of the long-term effects of repetitive head impacts across various sports and in both male and female athletes.

The greatest challenge in CTE research is the identification and validation of any potential biomarkers and their correlation with clinical features and post-mortem neuropathological changes. This is particularly important, given the lack of specificity regarding the current clinical criteria. Therefore, the establishment of our biobank and brain bank for storing blood, CSF, and brain tissue is a big advantage. The NEwTON brain bank is the first tissue repository focused on traumatic brain injury and CTE in the Netherlands. Participants will have the opportunity to donate their brain to the NEwTON brain bank in order to correlate the data we obtain within the study with a definite postmortem diagnosis of CTE.

Careful considerations have been made regarding the methodology of this study, but some decisions about the study population can be debated. The study population consists of symptomatic former contact sports athletes and does not include asymptomatic athletes; thus, the results cannot be used to determine the risk for CTE in this population. However, NEwTON has an exploratory nature, and its purpose is to find, identify, and phenotype patients with possible CTE. Therefore, the current protocol specifies that the former athletes to be included must have cognitive or behavioral symptoms. Additional subgroups may be added to the NEwTON study in the future as comparison population, in particular asymptomatic athletes. Important to mention is that the study procedures within the NEwTON study have significant overlap with the procedures within the Amsterdam Dementia Cohort [36]. Therefore, it will be possible to compare data of the NEwTON cohort with the Amsterdam Dementia Cohort and will allow researchers to compare participants with possible CTE to relatively young, well-phenotyped patients with Alzheimer’s disease or other type of dementias.

Another point of discussion is that NEwTON does not include tau PET scans as part of the research methods. PET scans that detect tau pathology in vivo have rapidly evolved in the last decades, but until now, this method has achieved little success in the detection of CTE-related tau pathology [25, 26, 37]. Current tau tracers have specifically been developed for Alzheimer’s disease and may not sufficiently bind to the tau structures found in CTE. It is hoped that CTE-specific tau tracers will become available in the future. If so, they can be validated in the NEwTON study at a later stage. With the aforementioned in mind, a unique MRI protocol has been included in the NEwTON study. Although DTI, fMRI, and QSM have shown promise as clinical tools to detect the microstructural changes that are thought to occur in TBI, further studies are needed to validate these techniques for CTE [38]. Another important fact to report is that these MRI scans are currently only acquired at baseline. Follow-up MRI scans may be added to the study protocol in the future, in order to obtain valuable information about MRI changes over time in individuals at risk for CTE.

## Conclusions

The NEwTON research project will assemble a unique cohort of male and female participants at risk for CTE, caused by various sources of repetitive head injury. Future results from the NEwTON study will contribute to current knowledge of CTE, by providing new evidence on clinical features, potential biomarkers, and disease progression. This study is expected to be a primary explorative base and designed to share data with other CTE-related cohorts worldwide. Sub-studies may be added in the future with this cohort as backbone

## Data Availability

Future datasets used and/or analyses during the study will be available from the corresponding author on reasonable request.

## Abbreviations

AD: Alzheimer’s disease
aβ: Amyloid beta
BNE: Brain Net Europe
BOLD: Blood-oxygenation-level dependent
CCI-20: Cognitive Change Index—20 item
CSF: Cerebrospinal fluid
CTE: Chronic traumatic encephalopathy
DIAGNOSE CTE: Diagnostics, Imaging, and Genetics Network for the Objective Study and Evaluation of Chronic Traumatic Encephalopathy
DTI: Diffusion tensor imaging
DWI: Diffusion-weighted images
FAB: Frontal Assessment Battery
FBI: Frontal Behavioral Inventory
FLAIR: Fluid-attenuated inversion recovery
fMRI: Functional magnetic resonance imaging
FSL: FMRIB Software Library
GDPR: General Data Protection Regulation
GDS: Geriatric Depression Scale
LP: Lumbar puncture
MADRS: Montgomery Asberg Depression Rating Scale
MMSE: Mini-Mental State Examination
MOCA: Montreal Cognitive Assessment
MRI: Magnetic resonance imaging
NBB: Netherlands Brain Bank
NEwTON: NEurodegeneration: Traumatic brain injury as Origin of the Neuropathology
OSU-TBI-ID: OHIO State University TBI Identification Method
PET: Positron emission tomography
p-tau: Hyperphosphorylated tau
QSM: Quantitative susceptibility mapping
TBI: Traumatic brain injury
TDP-43: TAR DNA-binding protein-43
TES: Traumatic encephalopathy syndrome
UMC: University Medical Centers
UPDRS: Unified Parkinson’s disease rating scale
VUmc: Vrije Universiteit medical center
WMA: World Medical Association
